# The influence of reading ability on subsequent changes in verbal IQ in the teenage years

**DOI:** 10.1016/j.dcn.2013.06.001

**Published:** 2013-06-25

**Authors:** Sue Ramsden, Fiona M. Richardson, Goulven Josse, Clare Shakeshaft, Mohamed L. Seghier, Cathy J. Price

**Affiliations:** Wellcome Trust Centre for Neuroimaging, UCL, London WC1N 3BG, UK

**Keywords:** IQ, Reading, Literacy, MRI, Neuroimaging, Adolescence

## Abstract

Intelligence Quotient (IQ) is regularly used in both education and employment as a measure of cognitive ability. Although an individual's IQ is generally assumed to stay constant across the lifespan, a few studies have suggested that there may be substantial variation at the individual level. Motivated by previous reports that reading quality/quantity has a positive influence on vocabulary acquisition, we hypothesised that reading ability in the early teenage years might contribute to changes in verbal IQ (VIQ) over the next few years. We found that good readers were more likely to experience relative improvements in VIQ over time, with the reverse true for poor readers. These effects were largest when there was a discrepancy between Time 1 reading ability and Time 1 VIQ. In other words, VIQ increases tended to be greatest when reading ability was high relative to VIQ. Additional analyses supported these findings by showing that variance in VIQ change associated with Time 1 behaviour was also associated with independent measurements of brain structure. Our finding that reading in the early teenage years can predict a significant proportion of the variance in subsequent VIQ change has implications for targeted education in both home and school environments.

## Highlights

• Reading ability predicts subsequent changes in verbal IQ (VIQ) in teenagers.
• The effect of reading was observed on all VIQ subtests, except arithmetic.
• VIQ changes were larger when there was a discrepancy between VIQ and reading.
• The effect of reading on VIQ changes was validated by structural brain changes.

## 1 Introduction

For many decades, cognitive ability has been assessed using the Intelligence Quotient (IQ) (Neisser et al., 1996), particularly in education and employment, and it has become an important means of assessing future potential. These IQ scores, which are typically expressed as an index where the population mean is 100 and the population standard deviation is 15, are well recognised in a range of environments and have entered common parlance. Of course, as the brain develops and matures over the lifespan, its ability to perform various cognitive tasks will change, so measures of IQ have always been based on age-adjusted norms in order to control for these typical development trajectories. Using IQ as a measure of future potential is necessarily based on the assumption that the adjustment for age controls for all the changes in cognitive abilities that might occur, with the exception of those caused by degenerative neurological conditions and/or traumatic brain injury. The age-adjusted measure is then expected to stay constant across the lifespan and can therefore be considered a fixed characteristic of the individual. This assumption has generally been regarded as being supported by the high correlation between IQ measured at one point in time and that measured at another: our own study (Ramsden et al., 2011), using data on a group of teenagers, showed a correlation of .792 between the individuals’ Full Scale IQ score at Time 1 and their corresponding score at Time 2, some 3.5 years later, while the average IQ of the group barely changed (i.e. 112 at Time 1 vs. 113 at Time 2).

Despite the apparent stability of IQ for groups, a few studies have suggested that there may be substantial variation in IQ at the individual level during childhood (Sontag et al., 1958), the teenage years (Ramsden et al., 2011, Ramsden et al., 2012) and into old age (Deary et al., 2012). Indeed, a correlation coefficient of .792 only accounts for 63% of the variation in score, leaving over one-third of the variance unexplained. Examination of the individual effects in Ramsden et al. (2011) show unexpectedly large falls in IQ in some individuals (up to 18 points) and unexpectedly large increases in others (up to 21 points). As IQ is based on an age-adjusted calculation, such changes do not represent absolute shifts in ability, but they do suggest that the individual's ability is changing relative to his/her peers. Support for this hypothesis came from neuroimaging data that found measured changes in IQ were linked to changes in the structure of the brain, with different brain areas being associated with changes in verbal IQ (VIQ) and non-verbal IQ (Performance IQ or PIQ).

The observation that changes in measured IQ reflect genuine changes in cognitive ability motivates further investigations into whether future IQ changes can be predicted from initial performance and what causes these changes to occur. As a first step in responding to these questions, we extended our analysis of the teenagers previously reported in Ramsden et al., 2011, Ramsden et al., 2012 to investigate whether reading performance at Time 1 predicted which individuals were likely to change their VIQ between Time 1 and Time 2. Our interest in reading ability was based on several previous studies that reported a positive influence of reading quality or quantity on vocabulary acquisition (Cunningham and Stanovich, 1997, Ferrer et al., 2010, Nagy et al., 1985, Stanovich and Cunningham, 1993, Swanborn and de Glopper, 1999). As vocabulary is one of the VIQ subtests, then one might expect that better reading will increase vocabulary which in turn increases VIQ. The relationship between reading ability and VIQ is also reflected by cross-sectional correlations at a single time point (Wechsler, 1992, p. 99; Wechsler, 1998, p. 161). As a consequence of these cross sectional correlations, reading ability at one time point can correlate with VIQ at another time point, particularly when standardised measures are used that have anticipated and corrected for expected age-related changes. However, our interest here is in whether relative rises and falls in (age-adjusted) VIQ could be predicted by reading ability at Time 1. This could occur if the development of performance on VIQ tests was accelerated between Time 1 and Time 2 in early readers but delayed in late readers. It was also possible that the influence of good or poor reading at Time 1 on subsequent VIQ changes (Time 2 − Time 1) might depend on VIQ at Time 1, since good reading ability and high verbal skills might be super-additive in effect (i.e. provide an accelerated additive boost), with the reverse true for poor reading and poor verbal skills.

## 2 Materials and methods

This study was approved by the Joint Ethics Committee of the Institute of Neurology and the National Hospital for Neurology and Neurosurgery, London, UK.

### 2.1 Participants

Our participants were the same sample of 33 teenagers whose longitudinal data were reported in Ramsden et al., 2011, Ramsden et al., 2012, where full details can be found. In brief, participants were 33 healthy teenagers (19 males, 14 female) aged between 12 and 16 years (mean 14.1) when tested at Time 1 and 15 and 20 years (mean 17.7) when tested at Time 2. The participants received normal schooling. No special experimental training was provided in advance of either testing round, and participants were not told that they would form part of a longitudinal study, so they had no experimentally-driven motivation to practice these skills in the time period between tests.

### 2.2 Behavioural assessments

VIQ was assessed with the Wechsler Intelligence Scale for Children (WISC-III; Wechsler, 1992) at Time 1 and the Wechsler Adult Intelligence Scale (WAIS-III; Wechsler, 1998) at Time 2, while Reading data were taken from the Wechsler Objective Reading Dimensions (WORD; Rust et al., 1993) at Time 1 and the Wide Range Achievement Task (WRAT; Wilkinson, 1993) at Time 2. It was necessary to use different tests in order to ensure that the tests were age-appropriate for the individuals taking them. Raw scores on all tests were converted to age standardised scores (mean 100, standard deviation 15) using procedures in the published statistical manuals for each test which are based on relatively large samples of data.

Given the careful standardisation of the tests there is no major reason to doubt the values achieved, in spite of the use of different tests at each time point. The statistical manual for the WAIS reports a correlation coefficient of .88 for VIQ scores for a sample of 16 year olds tested on both the WISC and the WAIS (US versions). The WORD manual reports a correlation coefficient of .84 for its reading test and the WRAT-R (an earlier form of the WRAT3 used here). Critically, however, our analysis does not depend on the absolute values of the scores at either time, but on the relative difference between standardised scores, and hence on the change in cognitive performance (after controlling for normal development). Thus, a change from 100 at Time 1 to 110 at Time 2 is regarded as being the same as a change from 120 at Time 1 to 130 at Time 2 (in both cases a change of +10), and both are larger than a change from 90 at Time 1 to 95 at Time 2 (+5), or a fall from 120 at Time 1 to 110 at Time 2 (−10). Changes in score are therefore seen as falling on a continuous scale from the largest fall to the largest increase, regardless of the absolute score at either testing point. Similar arguments apply when we consider the discrepancy between Reading scores and VIQ scores: our analysis is based on a continuum of differences, rather than focusing on whether the Reading score was higher or lower than the VIQ score. In short, it is the relative change or difference across individuals that is important.

Full details of the participant scores are provided in Table 1. VIQ scores ranged from 84 to 139 at Time 1 (a range of 55) and from 90 to 150 at Time 2 (a range of 60). As reported in Ramsden et al. (2011), there was a high correlation between VIQ at Time 1 and VIQ at Time 2 (*r* = .809, *p* < .001) and there was no significant difference in the average scores at Time 1 and Time 2 (113 vs 116, *t*_(32)_ = −1.697, *p* = .099). However 21% of our sample changed their VIQ score by at least one population standard deviation (15 points) over the period, with changes ranging from −20 to +23 points.

**Table 1:** Details of individual participants.

ID	Time 1					Time 2					Change (Time 2 − Time 1)		
	Age	VIQ	PIQ	Read	Read minus VIQ	Age	VIQ	PIQ	Read	Read minus VIQ	VIQ	PIQ	Read
1	14.4	115	110	95	−20	18.3	95	97	106	11	−20	−13	11
2	13.3	109	112	97	−12	17.1	95	98	102	7	−14	−14	5
3	13.6	136	112	123	−13	17.1	123	114	117	−6	−13	2	−6
4	12.6	115	110	103	−12	16.0	104	111	102	−2	−11	1	−1
5	13.8	127	116	99	−28	17.3	119	98	106	−13	−8	−18	7
6	14.8	102	94	73	−29	18.3	96	109	82	−14	−6	15	9
7	14.1	108	109	102	−6	17.4	104	106	104	0	−4	−3	2
8	13.7	133	101	114	−19	17.1	130	114	112	−18	−3	13	−2
9	16.0	128	137	102	−26	19.4	125	124	102	−23	−3	−13	0
10	13.7	98	112	91	−7	17.6	95	102	104	9	−3	−10	13
11	12.8	92	96	96	4	16.3	90	104	99	9	−2	8	3
12	16.0	96	116	86	−10	19.5	94	110	102	8	−2	−6	16
13	13.0	100	90	104	4	16.6	100	95	103	3	0	5	−1
14	13.7	117	125	108	−9	17.2	117	113	102	−15	0	−12	−6
15	14.4	91	97	73	−18	18.2	91	95	82	−9	0	−2	9
16	15.6	102	119	97	−5	18.9	102	102	106	4	0	−17	9
17	13.8	120	97	97	−23	17.3	121	114	117	−4	1	17	20
18	13.1	127	115	104	−23	16.6	131	111	108	−23	4	−4	4
19	13.6	137	105	117	−20	17.2	142	107	121	−21	5	2	4
20	14.8	108	110	106	−2	18.3	113	107	100	−13	5	−3	−6
21	13.8	121	109	111	−10	17.4	128	110	117	−11	7	1	6
22	13.5	84	74	97	13	17.2	91	83	97	6	7	9	0
23	14.4	98	97	89	−9	17.7	106	100	91	−15	8	3	2
24	14.1	101	88	79	−22	17.5	110	104	93	−17	9	16	14
25	13.4	139	115	123	−16	16.9	150	124	123	−27	11	9	0
26	13.5	131	112	117	−14	17.1	142	114	118	−24	11	2	1
27	16.5	117	121	111	−6	20.2	128	116	120	−8	11	−5	9
28	13.3	129	118	124	−5	16.9	144	117	127	−17	15	−1	3
29	14.3	113	124	117	4	17.8	130	113	110	−20	17	−11	−7
30	14.1	91	105	97	6	17.9	108	105	115	7	17	0	18
31	13.1	120	103	121	1	16.4	138	85	118	−20	18	−18	−3
32	16.3	104	101	111	7	19.8	127	104	108	−19	23	3	−3
33	15.5	110	103	109	−1	18.9	133	117	110	−23	23	14	1
Av	14.1	112.7	107.7	102.8	−9.9	17.7	115.8	106.8	106.8	−9.0	3.1	−0.9	4.0
SD	1.0	15.1	12.3	13.5	11.0	1.0	18.0	9.6	10.8	11.9	10.6	10.2	7.1

Participants are presented in order of increasingly positive VIQ change (Time 2 minus Time 1). Scores for VIQ and PIQ are from the WISC at Time 1 and the WAIS at Time 2. Reading scores are from WORD at Time 1 and WRAT at Time 2. All these tests have a population mean of 100 and a population Standard Deviation of 15. The “Av” and “SD” rows show the sample mean and standard deviation respectively. Age is shown in years. There is no significant difference between mean Time 1 and Time 2 scores for VIQ, PIQ and Reading minus VIQ; the difference between Time 1 Reading and Time 2 Reading is statistically significant (*t*_*(*32)_ = −3.204, *p* = 003).

As has been found in other studies (see Section 1), Reading and VIQ scores were correlated at a point in time (Time 1: *r* = .710, *p* < .001; Time 2: *r* = .769, *p* < .001) with no difference in the degree of correlation between Time points (after Fishers transformation, *z* = −.680, *p* = .248 one-tailed). As a result, Reading and VIQ scores were also coupled across time because Reading at Time 1 predicted VIQ at Time 2 (*r* = .802, *p* < .001 two tailed) and VIQ at Time 1 predicted Reading at Time 2 (*r* = .676, *p* < .001 two-tailed).

The average discrepancy between an individual's Reading and VIQ scores (Reading minus VIQ) at Time 1 (mean = −9.9, standard deviation = 11.0) and Time 2 (mean = −9.0, standard deviation = 11.9) were not significantly different (*t*_(32)_ = −.359, *p* = .722). However, the correlation between the discrepancy at Time 1 and Time 2 across participants was not significant (*r* = .298, *p* = .092).

### 2.3 Structural brain imaging

Data acquisition and pre-processing of the images, as detailed below, was identical to that reported in Ramsden et al. (2011).

#### 2.3.1 Data acquisition

At both time points (Time 1 and Time 2), structural brain images were acquired from the same Siemens 1.5T Sonata MRI scanner (Siemens Medical Systems). The images were acquired using a T1-weighted modified driven equilibrium Fourier transform sequence with 176 sagittal partitions and an image matrix of 256 × 224, yielding a final resolution of 1mm3 (repetition time, 12.24 ms; echo time, 3.56 ms; inversion time, 530 ms).

#### 2.3.2 Image processing

Pre-processing of the 66 structural images (33 participants, 2 time points), was conducted in SPM8 (http://www.fil.ion.ucl.ac.uk/spm) with the DARTEL toolbox to segment and spatially normalise the brains into the same template. DARTEL uses a more sophisticated registration model than previous approaches implemented in the SPM software (Ashburner, 2009). Structural images were first segmented in native space into grey and white matter. Grey and white matter images from a total of 172 scans in our database (including the 33 participants who took part in this study) were imported into DARTEL format. A template brain was then created in DARTEL using default parameter settings predefined within SPM8. This process iteratively matches selected images to a template generated by their own mean. There were six outer iterations, each consisting of three inner iterations. Over outer iterations, the parameter settings were as follows: mu = [4,2, 1, 0.05, 0.25, 0.25], lambda = [2,1, 0.5, 0.25, 0.125, 0.125], id = 1e − 006, time steps = [1,1,2,4,16,64], smoothing = [16,8, 4, 2, 1, 0.5]. The resulting flow fields containing deformation information generated by this process were then used to spatially normalise grey and white matter images to Montreal Neurological Institute (MNI) space.

Following segmentation and normalisation, the resulting images can be either modulated or unmodulated. Modulated images are corrected for the final volume of the surrounding area as measured by the degree of local compression in the normalisation process (i.e., the Jacobian determinates of the deformation). For example, if a brain area was exactly half the size of that in the template, then normalisation would require doubling its volume, thereby distributing the intensity of each voxel over twice as much space, and effectively halving each voxel's value. In this case, modulation would correct for this ‘halving’, by effectively multiplying each voxel by two (Mechelli et al., 2005). In this way, the voxels in modulated images provide an absolute measure of regional volume (Ashburner, 2009). By contrast, unmodulated images are not adjusted for volume, and each voxel provides an estimation of the likelihood the tissue is grey matter relative to white matter and other types of tissue. Crucially, the results of VBM studies often depend on whether modulated or unmodulated images are used. In our previous language studies, for instance, we found our VBM analyses were more sensitive and more consistent across subject groups when we used unmodulated images than when we used modulated images (Grogan et al., 2009, Lee et al., 2007, Mechelli et al., 2004, Richardson et al., 2010). This suggests that the effects are in the tissue density rather than volume. The resulting images were then smoothed using a Gaussian kernel with an isotropic full-width of 8 mm at half maximum.

It is worth noting that our approach to longitudinal analysis differs from the deformation-based morphometry (DBM) method referred to in Yushkevich et al. (2010) which can introduce bias because it involves pre-specification of the baseline image, the follow-up image, and the structure of interest. Our approach is not subject to such bias because it involves independent segmentation of whole brain images at each time point, and uses the difference in the two segmentations as the measure of interest.

#### 2.3.3 Statistical analysis

The relationship between change in VIQ and change in brain structure was investigated by entering the appropriate pre-processed images into within-subject paired *t*-tests, with change in VIQ, change in PIQ and year of scan as covariates. PIQ was included in order to isolate brain regions that were more related to verbal than non-verbal IQ. For both region identification and data extraction, we used a “leave-one-out” approach (see Martens and Dardenne, 1998) to ensure that the participants used to select the voxel of interest were independent of the participants used to extract the data for further analyses (Ramsden et al., 2012, Price et al., 2013). This approach has proved to be the preferred approach for quantifying out-of-sample effect sizes in our sample (Price et al., 2013). It involved a series of 33 statistical analyses that each included all but one of the 33 participants. We then extracted the change in grey matter density for each participant at the peak voxel in the analysis from which they were excluded. This process was repeated for each of the 33 participants, yielding unbiased estimates of grey matter density change for each individual in the sample. Notably, (i) in each of the 33 analyses, the peak voxel associated with VIQ change in the left motor speech area was identical to that previously reported (Ramsden et al., 2011; *x* = − 49, *y* = − 9, *z* = + 30) and (ii) in 32/33 analyses, the observed effect sizes at this voxel were significant (*p* < 0.05 in height) after family-wise error correction for multiple comparisons across the whole brain. Therefore, the data extracted using the leave-one-out procedure was identical to that used in Ramsden et al. (2011).

### 2.4 Predicting the change in VIQ from Time 1 behavioural data

Analyses of behavioural data were carried out in SPSS. This involved a correlational analysis of the VIQ change scores for each individual (Time 2 VIQ minus Time 1 VIQ) with (a) Time 1 Reading and (b) Time 1 VIQ. Using a 2-Step hierarchical multiple regression we then tested how well VIQ change was predicted by (c) Time 1 Reading after factoring out VIQ change associated with Time 1 VIQ; and (d) repeated this analysis, replacing Time 1 Reading scores with Time 1 PIQ. Finally we used 3-Step regression analyses that included (e) Time 1 VIQ and Time 1 PIQ prior to the inclusion of Time 1 Reading in order to predict VIQ change; and (f) repeated this analysis 5 times after replacing VIQ change and Time 1 VIQ with the corresponding scores from one of the 5 VIQ sub-tests that were common to both the WISC and the WAIS (see Table 2 and Fig. 2).

**Table 2:** Results of the 3-Step hierarchical multiple regression analyses: ability of Time 1 test scores to predict changes in test score.

Change in:	Step 1: predicting score change from Time 1 score on the same test				Step 2: predicting score change from Time 1 PIQ				Step 3: predicting score change from Time 1 reading score				
	Effect sign	*R*^2^ change	*F* change (1,31)	*p*	Effect sign	*R*^2^ change	*F* change (1,30)	*p*	Effect sign	*R*^2^ change	*F* change (1,29)	*p*	Minimum tolerance
VIQ	−	.003	.079	.781	−	.007	.215	.646	+	.303	12.780	.001	.427
Vocabulary	−	.039	1.251	.272	−	.018	.570	.456	+	.306	13.932	.001	.373
Similarities	−	.090	3.067	.090	+	.047	1.649	.209	+	.202	8.885	.006	.717
Arithmetic	−	.146	5.284	.028	−	.000	.001	.976	+	.072	2.686	.112	.604
Information	−	.382	19.180	< .001	+	.033	1.683	.204	+	.091	5.330	.028	.452
Comprehension	−	.347	16.477	< .001	−	.020	.934	.342	+	.190	12.414	.001	.784

Details of how well the change in scores [Time 2 − Time 1] on VIQ and its sub-tests was predicted by Time 1 performance. For each of the 6 analyses (rows in column 1), a three stage regression was performed. Step 1 tested the degree to which the change in score was predicted by Time 1 score on the same test (e.g. change in Vocabulary score predicted by Time 1 Vocabulary). Step 2 tested the degree to which the change in score was predicted by Time 1 PIQ, over and above the effect of Step 1. Step 3 tested the degree to which the change in score is predicted by Time 1 Reading score, over and above the effect of Steps 1 and 2. For each test (rows) and each step (columns), the table shows the direction of the effect (positive or negative), the change in *R*^2^ when each variable is inserted, the *F* value of the change (with degrees of freedom in brackets) and the significance of the *F* value (*p*). Minimum tolerance figures for Step 3 show the lowest tolerance figure for any of the three independent variables in the regression. These indicate that multicollinearity is not a serious problem in these regressions in spite of the correlations between some of the independent variables.

### 2.5 The relationship between behavioural data and brain structure

In SPSS, we tested whether the change in VIQ that was accounted for by Time 1 behaviour (a combination of Time 1 Reading and Time 1 VIQ) was independent or not from the change in VIQ that was accounted for by changes in brain structure over the same time period in the region identified in the leave one out VBM analyses described in Section 2.3 above. The area of interest was located in the left motor speech area, at the voxel that was most significantly associated with VIQ change, as described in Section 2.3, which provided independent estimates of grey matter density. The change in local grey matter (Time 2 minus Time 1) was put into a regression analysis predicting the change in VIQ, together with Time 1 behaviour (VIQ and Reading) in a series of hierarchical analyses. Shared and distinct variance between Time 1 behaviour and grey matter density change could then be identified.

## 3 Results

### 3.1 Predicting the change in VIQ from Time 1 behavioural data

**Fig. 1:**
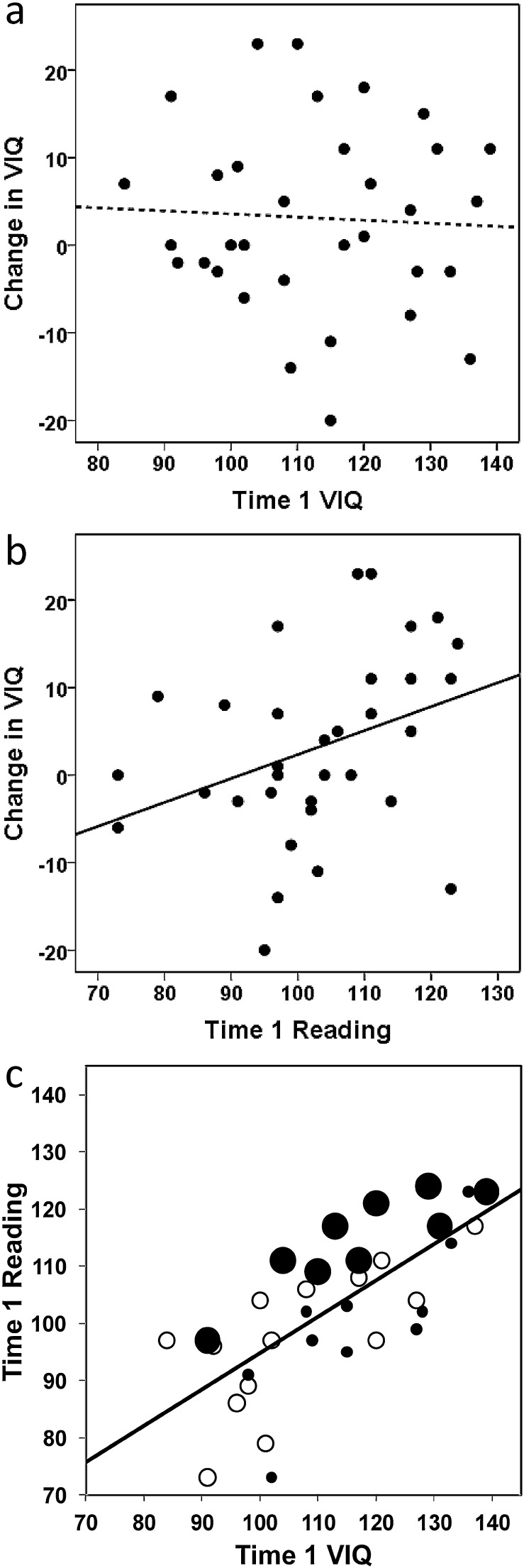
Correlation between the change in VIQ and Time 1 VIQ and Reading scores. The first two plots show the correlation between the change in VIQ [Time 2 − Time 1] and: (a) VIQ at Time 1 and (b) Reading at Time 1. Regression lines are shown as solid lines for significant effects and dotted lines non-significant effects (at 5%, two-tailed), (c) shows the correlation between Time 1 Reading and Time 1 VIQ, with the colour and size of the circles indicating the degree of subsequent VIQ change [Time 2 − Time 1]: large filled circles indicate a subsequent change in VIQ at or above the top quartile for our sample, small filled circles indicate a subsequent change in VIQ at or below the bottom quartile for our sample, and empty circles indicate a subsequent change in VIQ which falls within the inter-quartile range for our sample. The plot shows that the largest relative increases in VIQ (the large filled circles) tend to occur for those people in the sample whose Time 1 Reading score is highest relative to their Time 1 VIQ (i.e. above the regression line), regardless of their Time 1 VIQ, while the reverse tends to be the case for those with the smallest relative change in VIQ (the small filled circles).

Change in VIQ was significantly correlated with Time 1 Reading scores (*r* = .350, *p* = .046, two-tailed; see Fig. 1b) but was not significantly correlated with Time 1 VIQ (*r* = −.050, *p* = .781, two-tailed; see Fig. 1a). Consequently, VIQ change correlated significantly more strongly with Time 1 Reading than Time 1 VIQ (*t*_(30)_ = 2.413, *p* = .022 two-tailed). The effect of Time 1 Reading can be re-described in binary terms by categorising participants with high and low VIQ change (relative to the median for our sample = + 1) and high and low Time 1 Reading ability (relative to the median for our sample = standardised score of 103). This categorisation showed that high VIQ change was more likely within the high Time 1 Reading group (75%) than within the low Time 1 Reading group (24%). This difference in proportions was significant (*z* = −2.957, *p* = .003, two-tailed). Likewise, low VIQ change was more likely in those with low Time 1 Reading than high Time 1 Reading.

The 2-step hierarchical multiple regression analyses found that it was the combination of Reading and VIQ at Time 1 that provided the best prediction of VIQ change: while the correlations showed that 0.3% of the variance in VIQ change was explained by VIQ at Time 1 alone and 12.3% was explained by Reading at Time 1 alone, 30.3% was explained by a combination of both measures (overall *F* = 6.521, *p* = .004), with a negative coefficient on Time 1 VIQ (*β* = −.604, *t* − 2.787, *p* = .009) and a positive coefficient on Time 1 Reading (*β* = + .779, *t* = 3.596, *p* = .001). The advantage of including both VIQ and Reading in the regression analysis emerged because the largest changes in VIQ in our sample were observed in cases where the divergence between Time 1 Reading and Time 1 VIQ was largest, regardless of the initial value of VIQ. This is illustrated in Fig. 1c, where it is clear that the people with the largest relative improvements in VIQ (large filled circles) fall above the regression line, indicating that the divergence between their Time 1 Reading score and their Time 1 VIQ was higher than for other members of the sample. Importantly, it is also clear that those with the largest improvements in VIQ are drawn from across the range of Time 1 VIQ scores, but they are also not confined to the very best readers. Similarly, those with the smallest changes in VIQ are spread across the range of both VIQ and Reading scores.

**Fig. 2:**
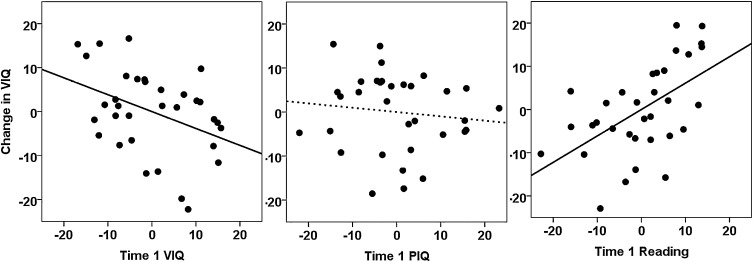
Partial plots showing relationship between change in VIQ and Time 1 test scores included in the third step of the hierarchical multiple regression analysis (see Table 2). Partial plots are shown for the Step 3 regression for the change in VIQ (first line of Table 2). They show the residuals of the dependent variable (change in VIQ) and each of the independent variables, when all are regressed separately on the remaining independent variables. These plots demonstrate that the effects are not driven by outlier effects.

The second 2-Step hierarchical multiple regression (replacing Time 1 Reading scores with Time 1 PIQ) demonstrated that the influence of Time 1 Reading was not a general result occurring simply because an additional variable had been added to the model, because Time 1 PIQ was not a significant predictor of VIQ change. Moreover, the 3-Step hierarchical multiple regression (including Time 1 VIQ, Time 1 PIQ and Time 1 Reading) showed that the inclusion of PIQ did not affect the predictive power of Time 1 Reading (see Table 2 and Fig. 2).

Finally, 5 additional 3-Step hierarchical regression analyses showed that Time 1 Reading significantly predicted the change in score in each of the sub-tests that are common to both the WISC and the WAIS, with the single exception of Arithmetic (see Table 2).

### 3.2 The relationship between behavioural data and brain structure

**Fig. 3:**
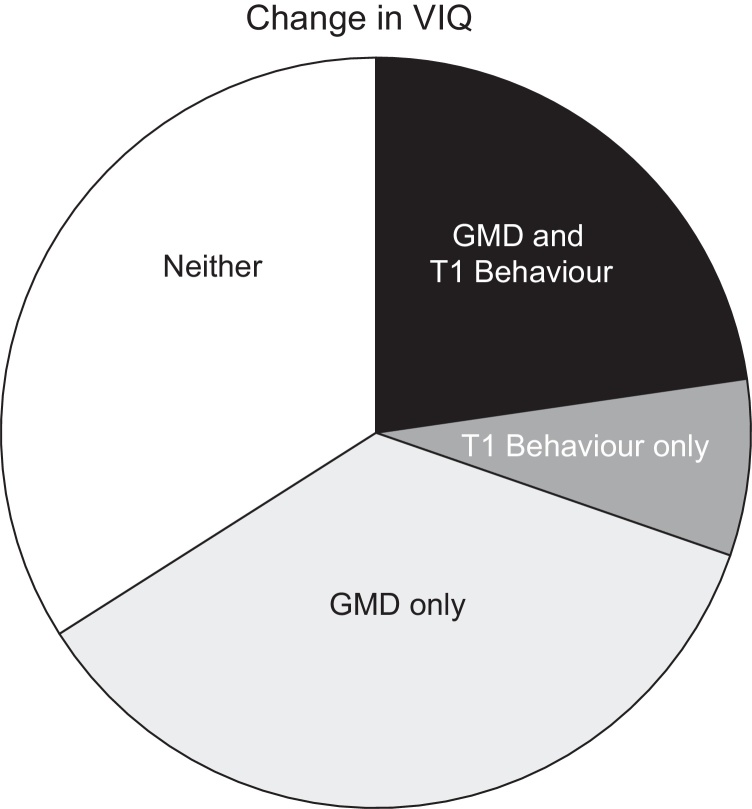
Allocation of total variance in VIQ change. The figure shows the predictors of the change in VIQ [Time 2–Time 1], and shows the relative contributions of the change in grey matter density and Time 1 Behaviour (Reading and VIQ entered separately into the equation). T1 = Time 1. GMD = the change in grey matter density [Time 2–Time 1]. Behaviour = Reading and VIQ as separate variables.

To validate the effect of Time 1 Reading ability on subsequent VIQ changes, we considered whether the change in VIQ that was accounted for by Time 1 behaviour (a combination of Time 1 Reading and Time 1 VIQ) was independent or not from the change in VIQ that was accounted for by changes in brain structure over the same time period. This change in brain structure was located in the left premotor cortex at MNI co-ordinates [−47, −9, +30] (Fig. 1, Ramsden et al., 2011) extending back, across the central sulcus, to the left somato-sensory cortex at MNI co-ordinates [−60, −15, +33] (Table 2, Price et al., 2013). We found that 75% of the variance in VIQ change that was associated with Time 1 behaviour was also associated with local grey matter change in this region (see Fig. 3). This shows that reading at Time 1 predicts variance in VIQ change that is likely to be genuine (rather than measurement error) because it is cross-validated by changes in brain structure. In addition, this analysis suggests the influence of other (unaccounted for) factors on VIQ change because 36% of the variance in VIQ change that was associated with grey matter density change was not associated with Time 1 behaviour (Fig. 3). Consequently, grey matter density change, in the left motor speech area, was not significantly correlated with either Time 1 Reading (*r* = .216, *p* = .227) nor Time 1 VIQ (*r* = −.077, *p* = .669).

### 3.3 Other possible effects

We checked that, in spite of the age-standardisation of scores, age at Time 1 was not having an effect on the analysis. Partialling Time 1 age out of the correlations did not change the significance of the effects: VIQ change was significantly predicted by Time 1 Reading (*r* = .698, *p* < .001), but not by Time 1 VIQ (*r* = −.008, *p* = .967).

We also checked whether gender had an effect. Males and females did not differ significantly in terms of Time 1 VIQ (*t*_(31)_ = −.167, *p* = .869); Time 1 Reading (*t*_(31)_ = −.686, p = .498); Time 1 VIQ minus Time 1 Reading (*t*_(31)_ = .613, *p* = .544); or VIQ change (*t*_(31)_ = .253, *p* = .802). There was also no significant difference between the sexes in the correlation between VIQ change and either Time 1 VIQ or Time 1 Reading.

Finally, we tested whether there was a correlation between Time 1 Reading and the change in non-verbal IQ (PIQ): this correlation was not significant (*r* = − .115, *p* = .526, two-tailed).

## 4 Discussion

The aim of this study was to identify factors that might predict changes in VIQ during the teenage years. Previously we demonstrated, with the same dataset, that changes in VIQ were accompanied by significant changes in brain structure, therefore providing independent evidence that VIQ changes were genuine rather than measurement error (Ramsden et al., 2011, Ramsden et al., 2012). In the current study we show that VIQ change was significantly predicted by Time 1 Reading, but not Time 1 VIQ. Moreover, we found the effect of reading was greatest when there was a discrepancy between Time 1 Reading and Time 1 VIQ. We validate these findings by showing that 75% of the variance in VIQ change that was associated with Time 1 behaviour was also associated with local grey matter change in the same motor speech area that was reported in Ramsden et al., 2011, Ramsden et al., 2012. These results, and their possible causal mechanisms, are discussed below.

The main finding is that VIQ change in the teenage years was significantly predicted by Time 1 Reading. We therefore confirmed our expectation that those with good initial reading ability were more likely to show relative improvements in VIQ than were those with poor initial reading. In contrast, Time 1 VIQ did not predict VIQ change. An additional and unexpected finding was that the effect of Time 1 Reading depended on Time 1 VIQ with the largest changes in VIQ observed for those in our sample for whom the divergence between Reading and VIQ was largest, regardless of the initial value of VIQ. This finding may be important for predicting that, for example, VIQ may improve if Time 1 Reading is high compared to Time 1 VIQ. Conversely, VIQ may fall if Time 1 Reading is low compared to Time 1 VIQ. In the following discussion we consider this finding prior to ruling out explanations relating to measurement error.

The influence of reading on subsequent VIQ illustrates that the development of different cognitive skills can progress along trajectories that (i) have different learning rates and (ii) interact with one another. In this context, our findings can be interpreted as follows: in early childhood, verbal skills are more likely to develop from oral communication than reading but, in most educated societies, verbal skills will be increasingly influenced by reading from the end of the first decade of life or early teenage years. The boost that reading gives to VIQ will therefore lag behind, rather than occur in synchrony with, the development of reading skills. Consequently, there may be a short lived discrepancy between Reading ability and VIQ scores that may only be detectable at a limited set of time points. In this context, our participants could, in theory, be classified into three different types: (1) Reading acquisition had already boosted VIQ by Time 1, resulting in no further boost to VIQ at Time 2; (2) Reading was good relative to VIQ at Time 1 with the subsequent boost to VIQ occurring between Time 1 and Time 2, which would result in a relative VIQ increase in our data set; (3) Reading ability was poor relative to VIQ at Time 1, limiting the development of verbal skills, possibly resulting in a decline in VIQ (remembering always that VIQ is age-adjusted, so a decline in VIQ does not imply a deterioration in verbal skills). Clearly, due to interpersonal differences in the timing of developmental trajectories, there are exceptions in our sample – for example individuals with relatively poor VIQ and Reading at Time 1 whose performance in both improves significantly by Time 2. This proposal could be tested by future studies that monitored Reading ability and VIQ over a longer time scale, with the expectation that individuals will vary in the age at which Reading ability influences VIQ. If this proposal is correct, then both reading and VIQ measures would need to be considered when estimating future VIQ scores from data collected in childhood or the teenage years.

In terms of how Time 1 Reading had its effect, our strongest prediction was that reading would influence subsequent vocabulary scores (Nagy et al., 1985, Swanborn and de Glopper, 1999) and this prediction was confirmed. In addition, we observed effects of Time 1 Reading on subsequent changes in the scores on the “similarities”, “information” and “comprehension” subtests contributing to VIQ, consistent with the widespread benefits that reading is known to have on a range of cognitive skills (Stanovich and Cunningham, 1993, Cunningham and Stanovich, 1997). The only score changes that were not significantly predicted by Time 1 Reading, in our study, were those from the arithmetic subtest.

The consistent effect of Time 1 Reading on each of the VIQ subtest scores, with the exception of arithmetic, suggests that reading ability might exert its beneficial effect on VIQ by enabling and motivating the acquisition of the verbal knowledge that is relevant to a wide range of tests (but not arithmetic). This virtuous circle or “Matthew effect” (Stanovich, 1986) leads to those who can read easily reading more, improving their reading comprehension through practice, acquiring more vocabulary and general knowledge, and thereby further easing the task of reading. By contrast, those who find reading slow or difficult are deterred from reading, do not reap the benefits that reading can bring, find that they fall further behind their class-mates in reading ability and knowledge levels, and are further discouraged from reading. Although this hypothesis explains why we found that more skilled readers show relative improvements in verbally-based skills (but not non-verbal skills), future studies are required to ascertain the learning mechanisms, for example, by segregating the influence of Time 1 reading comprehension from Time 1 decoding abilities. Future studies would also be necessary to determine whether external interventions might be able to influence this process, by, for example, enhancing reading skills at an appropriate developmental point in order to influence the eventual VIQ level.

Our finding that 75% of the variance in VIQ change that was associated with Time 1 behaviour was also associated with local grey matter change in the left premotor/somatosensory cortex may also provide clues to the mechanisms underlying changes in VIQ and reading scores. Previous studies have demonstrated that skilled readers show higher cerebral blood flow in the left premotor and somato-sensory cortex for reading relative to object naming (Bookheimer et al., 1995, Price et al., 2006). A speculative explanation, requiring future investigation, is that skilled readers engage in more somatosensory (or auditory) monitoring of their speech output during reading than object naming, to reduce errors when speech production is rapid or subject to conflicting phonological codes from lexical and sublexical processing. Those with better reading/higher VIQ may be become less susceptible to speech errors. Critically, we are not suggesting that this is the only explanation of the brain changes associated with VIQ change in the left premotor cortex because 36% of the variance in VIQ change that was associated with grey matter density change was not associated with Time 1 behaviour (Fig. 3).

We now turn to consider and reject some other possible explanations of our findings, which relate to various kinds of measurement error. The simplest form of measurement error is random noise. When there are two measurements, random noise may be greater at one time point than another. The expectation is that extreme values at Time 1 will be closer to the mean at Time 2 (known as “*regression to the mean”*) whereas extreme values at Time 2 will be further from the mean than at Time 1 (known as “*dispersion from the mean”*). Although these effects may, theoretically exaggerate the size of the effects in our data, they cannot explain the significance of the effects per se, which was validated by independent measurements of brain structure in Ramsden et al., 2011, Ramsden et al., 2012.

A second form of measurement error would be more systematic and might occur because of deficiencies in a behavioural test used at one time point or the other. All the tests used in this study have been extensively used and are well tested and documented, so although there is no particular reason to suppose that this might be the case, we can still consider it as a theoretical possibility. For example, it is possible that one of the two IQ tests used might be less discriminatory at the extremes than the other: if the Time 1 test (WISC) was less discriminatory than the Time 2 test (WAIS), then this might lead to dispersion from the mean (as the true performance of the Time 1 outliers was measured better in the second test), while regression to the mean would result from the opposite pattern. Our data provides no support to these hypotheses because the range of scores is similar on both occasions (see Section 2.2), while there is no significant correlation between the Time 1 VIQ score and the change in VIQ (see Section 3.1) as might have been expected (positive in the case of dispersion from the mean or negative in the case of regression to the mean). It is also not clear why this sort of measurement error would have generated a correlation between Time 1 Reading and the change in VIQ which could be observed across the full range of Time 1 VIQ scores (Fig. 1c).

An argument which could potentially explain the association with Reading observed here would be that, for some reason, measured Reading ability was a better indicator of true VIQ than was measured VIQ at Time 1, but not at Time 2. This might possibly result from greater distractibility of younger adolescents than older adolescents when undertaking VIQ tests (novel tasks) but not when undertaking reading tasks (a more commonplace task). Again we are not aware of any external evidence to support this hypothesis given the tests used, but we also note that our data does not provide any support. This hypothesis would suggest a weaker correlation between Reading and measured VIQ at Time 1 than Time 2, but we did not observe this (see Section 2.2). It might also lead us to expect that the average discrepancy between an individual's Reading and VIQ scores (Reading minus VIQ) would be greater at Time 1 than Time 2 but this was also not the case (see Section 2.2). Moreover, if the effect of Reading was the consequence of measurement error, then we would not expect the variance in VIQ change that is explained by Time 1 Reading to be shared with the variance in VIQ change that was explained by grey matter density in Ramsden et al., 2011, Ramsden et al., 2012. To the contrary, however, we found that 75% of the variance in VIQ change that was accounted for by the combination of Time 1 Reading and Time 1 VIQ was also associated with a change in grey matter density in the left motor speech area (see Section 3.2). It is still possible that the remaining 25% of variance in VIQ change that was accounted for by Reading but not brain structure may have been measurement error. Alternatively, future studies may find that this variance can be explained by other variables, for example, grey or white matter changes in other brain areas. It is also relevant to note at this point that although Reading at Time 1 had a significant influence on VIQ change, 36% of VIQ change that was accounted for by grey matter was not accounted for by Reading at Time 1. This simply emphasizes that Reading is likely to be one of several factors influencing cognitive development in the teenage years.

Having discussed and dismissed a number of theoretical forms of measurement error, it is worth noting that regression to the mean is not necessarily the result of measurement error. As discussed in Section 1, IQ scores are calculated on the basis of a typical developmental path, and this study considers a period in the lives of adolescents when abilities are developing rapidly. Individuals whose developmental trajectory differs from the typical pathway will find that their position relative to their peers changes over time. So, for example, Table 2 shows that, for some VIQ sub-tests, there is a significant negative relationship between Time 1 score and the corresponding score change, and this is also true for VIQ if Time 1 Reading is controlled for (Section 3.1). This is likely to indicate that: (i) some participants were early developers (high VIQ at Time 1 relative to their ultimate potential) who later fell back relative to their peers at Time 2; and/or (ii) other participants were late developers (low VIQ at Time 1 relative to their ultimate potential) who caught up with their peers by Time 2. We have proposed that Reading ability plays an influence in the early/late development of VIQ, but also emphasise again that there are likely to be many environmental and genetic factors influencing both reading and VIQ development.

## 5 Conclusions

While previous studies have shown that reading and VIQ scores correlate over time (Ferrer et al., 2010), our data provide two novel conclusions. First we show that, despite the coupling of reading and VIQ, reading ability in the early teenage years predicts the relative rise or fall of VIQ in later teenage years better than does early VIQ. Our explanation is that good reading skills have wide-ranging benefits on the subsequent development of verbal skills. Second, we have found indications that the influence of reading ability (good or poor) on VIQ change is most likely when there is a discrepancy between age-standardised scores for Reading and VIQ at Time 1. These novel findings were validated by showing that the influence of reading on VIQ change is substantially represented by corresponding changes in grey matter density measured from structural brain scans (which are independent of behaviour).

Future studies are required to investigate these effects further (over longer timescales and with larger samples), and to identify further factors that contribute to changes in VIQ (and non-verbal IQ), as well the causes of the discrepancy between reading and VIQ scores. For example, is the correlation between Reading at Time 1 and subsequent VIQ change driven by genetic factors, environmental factors or a combination of the two? One way to assess the impact of environment would be to introduce special programmes that determine how experimentally facilitated gains in reading influence subsequent verbal IQ at different ages and in populations with different cognitive abilities. An understanding of this relationship may be important for maximising the performance of all young people but, in particular, critical for maintaining VIQ in those who suffer from dyslexia. For example, supplementing education of these individuals with audiobooks may help to minimise the impact of poor reading ability on verbal knowledge that is typically acquired through reading.

## Conflict of interest statement

None declared.
